# TRPV1: Role in Skin and Skin Diseases and Potential Target for Improving Wound Healing

**DOI:** 10.3390/ijms22116135

**Published:** 2021-06-07

**Authors:** Michelle D. Bagood, R. Rivkah Isseroff

**Affiliations:** 1 Department of Dermatology, School of Medicine, UC Davis, Sacramento, CA 95816, USA; mdbagood@ucdavis.edu; 2 Dermatology Section, VA Northern California Health Care System, Mather, CA 95655, USA

**Keywords:** TRPV1, wound healing, skin, keratinocytes, pilosebaceous unit, nociceptors

## Abstract

Skin is innervated by a multitude of sensory nerves that are important to the function of this barrier tissue in homeostasis and injury. The role of innervation and neuromediators has been previously reviewed so here we focus on the role of the transient receptor potential cation channel, subfamily V member 1 (TRPV1) in wound healing, with the intent of targeting it in treatment of non-healing wounds. TRPV1 structure and function as well as the outcomes of TRPV1-targeted therapies utilized in several diseases and tissues are summarized. In skin, keratinocytes, sebocytes, nociceptors, and several immune cells express TRPV1, making it an attractive focus area for treating wounds. Many intrinsic and extrinsic factors confound the function and targeting of TRPV1 and may lead to adverse or off-target effects. Therefore, a better understanding of what is known about the role of TRPV1 in skin and wound healing will inform future therapies to treat impaired and chronic wounds to improve healing.

## 1. Introduction

**Figure 1:**
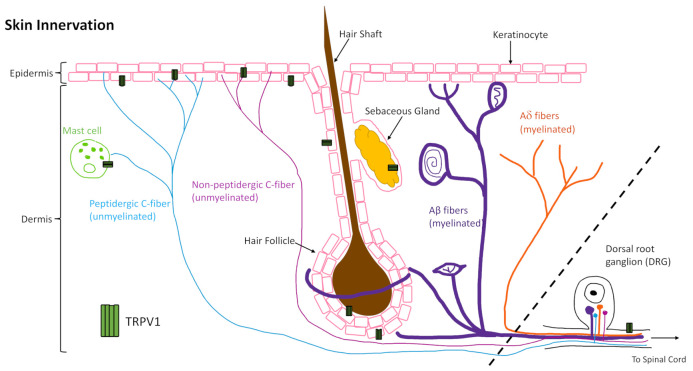
Sensory nerves of the skin and transient receptor potential cation channel, subfamily V member 1 (TRPV1) expression. The skin is innervated by sensory nerves originating from the dorsal root ganglion (not in the skin as noted by the dashed line) that can be categorized as myelinated (Aβ- and Aδ-fibers) or unmyelinated (C-fibers). C-fibers can be further characterized as peptidergic or non-peptidergic and terminate in the epidermis. C-fibers, mast cells, keratinocytes, sebocytes, and hair follicles, to name a few, have been shown to express TRPV1 (green tetramer, not to scale).

The skin is innervated by afferent sensory nerves, to detect and distinguish between innocuous and noxious stimuli, and efferent autonomic nerves, to maintain barrier tissue homeostasis [1]. The sensory nerves of the skin that originate from the dorsal root ganglion (DRG) can be further categorized as dermal myelinated Aβ- and Aδ-fibers, which make up about 80% of DRG afferents, and unmyelinated C-fibers that make up the other 20% and are present in the dermis and epidermis (Figure 1) [2]. The C-fibers are either peptidergic or non-peptidergic based on the presence or absence of neurotransmitter peptide expression such as substance P or calcitonin gene related-peptide (CGRP) that signal in an autocrine and paracrine manner to elicit downstream effects [3]. About 70% of the unmyelinated C fibers are classified as C-polymodal nociceptors, which respond to various trophic stimuli determined by their membrane protein expression, such as calcium and sodium channels (e.g., TRPV1 and Na_V_1.8) [2]. Classification based on neurotransmitter expression does not align with classification based on membrane protein expression as there is overlap in some fibers but not in others. This is an important nuance to consider for the various gain and loss of function models utilized in the studies reviewed here. Moreover, C-fibers have been shown to interact with keratinocytes, mast cells (Figure 1), Langerhans cells, blood vessels, and other cells of the skin to perform efferent functions through release of neuropeptides [4,5,6,7,8,9,10,11,12]. These neuronal mechanisms govern normal skin physiology but, when dysregulated, can contribute to abnormal cutaneous manifestations.

One area where integration of neuronal signaling is critical is in tissue repair, where neuroimmune mechanisms signal for cellular responses that contribute to the repair mechanism. The global role of innervation and neuromediators in cutaneous wound healing has been reviewed previously [13,14,15]. Therefore, this review focuses on a well-characterized channel, the transient receptor potential cation channel, subfamily V member 1 (TRPV1, also known as the vanilloid receptor 1 (VR1) and transient receptor potential vanilloid 1), that is highly expressed by cutaneous peripheral sensory nerves, as well as central nerve endings in the DRG, and in varying degrees on other skin and immune cells (Figure 1). Understanding what is already known about its role in wound healing, and how TRPV1-targeted therapies work in skin, as well as other tissues, may inform future therapies to improve healing in impaired and chronic wounds.

## 2. TRPV1 Structure and Function

**Figure 2:**
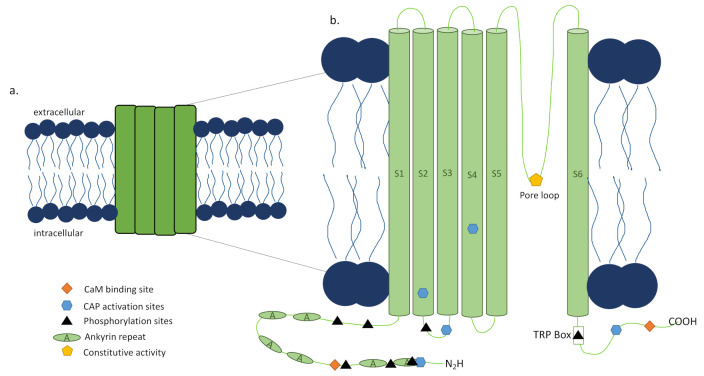
Transient receptor potential cation channel, subfamily V member 1 (TRPV1) structure. (**a**) The TRPV1 channel is a nonspecific ion channel expressed in the cell membrane made of four subunits with 838 amino acids per subunit, and homo- and heterotetrameric forms. When activated by capsaicin (CAP) or other stimuli, the dual gate pore opens and allows ion influx into the cell, with high relative permeability to calcium ions. (**b**) Each subunit of the TRPV1 channel is made up of six transmembrane domains. The pore loop is located between the S5 and S6 segments. Both the N and C termini are located intracellularly where they interact with CAP and phosphatases. The N terminus contains six ankyrin repeats, known as ankyrin repeat domain of TRPV1 (TRPV1-ARD), as well as a calmodulin (CaM) binding site. The C terminus contains the TRP box and a CaM binding site involved in stability and function. Adapted from [24] Figure 2.

The capsaicin activated TRPV1 channel was first discovered in rats by the Julius Lab in 1997 through cloning [16] and its human ortholog showed similar properties [17]. TRPV1 is a tetrameric ion channel [16], each subunit consisting of 838 amino acids (Figure 2a) [18], that has been found in homotetrameric and heterotetrameric forms [19,20], each with distinct functional properties [21]. The 3D structure of TRPV1 has been determined by electron cryomicroscopy showing that it is 150 Å tall and consists of two major regions [18]. The small region is 60 × 60 Å and 40 Å high, while the large region is 100 × 100 Å with a height of 110 Å with a basket-like mass containing a large opening in its center [18]. The N and C termini of each subunit of the channel are located intracellularly [16], with the N terminus playing a role in the channel’s sensitivity to activators [22] and the TRP box containing C terminus impacting channel stability and function (Figure 2b) [23,24]. Additionally, sensitization or desensitization of the TRPV1 channel can be achieved through the modulator protein calmodulin (CaM) depending on where it is bound to TRPV1 [23,24] or phosphorylation (Figure 2b) [24] of the channel by protein kinase C (PKC) [25], protein kinase A (PKA) [26], or calcium calmodulin-dependent kinase II (CaMKII) [27]. Activators of TRPV1 include not only capsaicin, the compound isolated from chili peppers that is responsible for their burning sensation, and other vanilloids and endovanilloids, but also noxious heat (>43 °C) [16], acidic conditions [28], divalent cations [29], and several animal toxins [30,31,32,33], though this list is still under investigation (reviewed by Fischer et al. [34]). These activators can act individually through distinct pathways as well as functionally coupled, making the channel activation polymodal and complex (reviewed in detail by Zheng [23]). Upon activation by a ligand, channel opening is achieved through a dual gate mechanism involving conformational changes in a selectivity filter and a lower gate [35]. Opening the TRPV1 channel permeabilizes the cell membrane to ions in a nonselective manner, with very high relative permeability to calcium ions [16]. The downstream signaling activated by the calcium influx is determined by the intracellular machinery present, as is the functional impacts of this signaling on the cell. To further complicate matters, there have been reports of alternative splicing of TRPV1, which can lead to nonfunctional channels or splice variant co-expression with TRPV1 that inhibit function [36]. Altogether, the multilayered intricacy of the TRPV1 channel, its activators, and the downstream effects of its activation make developing TRPV1-targeted therapy and the ultimate outcome of this therapy complex.

Prior to the discovery of the TRPV1 channel, capsaicin use to activate and desensitize sensory nerves became an active area of research in preclinical models in the 1970s [37], but the burning feeling and increased blood flow in skin after its application was observed by Hogyes a century before that in 1878 [38]. Indeed, TRPV1 activation by capsaicin forms the basis for a number of therapeutic and diagnostic approaches. However, the reversibility and extent of desensitization reached using capsaicin or its ultrapotent analog, resiniferatoxin (RTX), remained elusive [34], as shown by RTX being sensory nerve specific and not affecting TRPV1 expression in skin [39]. In 2000, the Julius lab created and characterized a TRPV1 knockout mouse (Trpv1^−/−^) that lacks exon 13, encoding the pore loop and transmembrane domain 6 of the channel, and does not respond to capsaicin [40]. The development of this mouse and other tools has allowed preclinical studies and made TRPV1 an appealing pharmacological target, though the outcome of the therapy is as varied as the conditions it is utilized to treat and the precise mechanisms are still under investigation.

## 3. Targeting TRPV1

The majority of TRPV1-targeted therapies are for the treatment of pain, however, more work is being done in the fields of urology, oncology, and dermatology, and recently in skin wound biology. TRPV1 channel agonists, such as capsaicin and RTX, were utilized to desensitize neurons and reduce neurogenic inflammation prior to the discovery of the specific channel they activated, and are still in use today to treat many types of pain [41]. Several antagonists and a few agonists have been developed and progressed to clinical trials in various tissues and pathologies [41,42]. In addition to direct activation or inhibition of TRPV1, extracellular hyaluronan (HA) was shown to stabilize the closed state of the TRPV1 channel, thereby reducing TRPV1-mediated firing in DRG neurons in vitro and reducing heat and capsaicin nocifensive responses after HA subcutaneous injection in vivo [43]. While there have been clear benefits demonstrated from TRPV1-targeted therapies, the complexity of the signaling pathways affected and the function of the channel itself have shown associated risks, making it imperative to weigh all these factors when pursuing these treatments.

### 3.1. Clinical Targeting of the TRPV1 Channel with Capsaicin to Treat Pain

While capsaicin is known as the molecule that gives peppers their burning sensation and causes pain, it has paradoxically been utilized to treat pain. It was observed that repeated or high doses of capsaicin produced analgesia after the initial pain subsided, called desensitization [44]. The mechanism of desensitization is not fully understood, but there are several proposed actions that are not mutually exclusive but are dependent on concentration and frequency of capsaicin application. One proposed mechanism is the depletion of neuropeptides in the sensory nerves expressing TRPV1 after activation by capsaicin, resulting in loss of sensitivity to stimuli by the neuron [45]. Another possible mechanism for the refractory state is the influx of calcium leading to calcium-dependent proteins acting on the channel to desensitize it. Rosenbaum and colleagues have shown that the cytosolic ankyrin repeat domain of TRPV1 (TRPV1-ARD), which sensitizes TRPV1 when bound to ATP, is bound by calmodulin (CaM) in a calcium-dependent manner to desensitize TRPV1 after capsaicin activation (Figure 2b) [46]. Finally, desensitization may be the result of capsaicin-induced cell death of TRPV1 expressing (TRPV1^+^) sensory neurons [47], most likely via caspase-dependent apoptosis and mitochondrial permeability [48], though there is evidence of caspase-independent cell death as well [49]. Furthermore, Menendez and colleagues showed that capsaicin-mediated analgesia is enhanced up to 30 days in inflammatory environments compared to control [50]. These are just a few of the studies that have begun to reveal the mechanisms underlying the analgesic effects of capsaicin, and future investigations will reveal more details of how they work individually and together. Not fully understanding desensitization has not stopped the use of capsaicin clinically to treat pain.

Capsaicin is available by prescription and over the counter in various forms to treat a wide range of pain conditions. Capsaicin cream is available in 0.025%, 0.075% and 0.1% concentrations and has been utilized to treat pruritus [51], postherpetic neuralgia [52], osteoarthritis [53], and diabetic neuropathy [54]. The application of topical capsaicin (0.075%) four times a day over a three-week period resulted in degeneration of sensory nerves and reduced sensitivity to all cutaneous stimuli, especially noxious heat and mechanical stimuli [55]. In addition to creams, capsaicin has been used in a nasal application to reduce the occurrence of cluster headaches [56] and a clinical trial has been conducted to test the effectiveness of capsaicin lozenges to treat mucositis caused by radiation therapy (NIH U.S. National Library of Medicine Clinicaltrials.gov. Available online: www.clinicaltrials.gov; NCT00003610 (accessed on 9 April 2021). The past success of capsaicin treatment for various forms of pain has led to continued investigation of its efficacy in ongoing clinical trials.

The majority of clinical trials are assessing the benefits of a capsaicin patch (NGX-4010 or trade name Qutenza) to treat neuropathic pain [41,57]. A capsaicin 8% patch was well tolerated and effective when used to treat post-traumatic and post-surgical neuropathic pain [58], post-herpetic neuralgia [59], HIV-associated distal sensory polyneuropathy [60], and peripheral diabetic neuropathy pain [61]. This patch is in trials to treat pain resulting from a variety of diseases, including HIV-associated neuropathy [62], herpes, surgery, amputation, cancer, and complex regional pain syndrome type 1 (NIH U.S. National Library of Medicine Clinicaltrials.gov. Available online: www.clinicaltrials.gov; NCT03794388, NCT01748435, NCT04704453, NCT00468390 (accessed on 9 April 2021). The 8% capsaicin Qutenza patch (Acorda Therapeutics, Ardsley, NY) and capsaicin topical liquids (1% and 5%) are in clinical trials to treat osteoarthritic pain (NIH U.S. National Library of Medicine Clinicaltrials.gov. Available online: www.clinicaltrials.gov; NCT03528369 (accessed on 9 April 2021), as well as the intraarticular injection of trans-capsaicin to treat osteoarthritic pain in the knee [63]. A 0.075% capsaicin gel has sufficiently treated acute back and neck pain [64] and also is in a phase III clinical trial to treat diabetic neuropathy (NIH U.S. National Library of Medicine Clinicaltrials.gov. Available online: www.clinicaltrials.gov; NCT03113448 (accessed on 9 April 2021). The use of capsaicin to target TRPV1 in pain has revealed the potential of focusing on this channel and opened the door to treating many other conditions via TRPV1. Understanding the successes and off-target effects of TRPV1-targeted treatments in other tissues and diseases reveals mechanisms that may be utilized in the skin, as well as cautions against unwanted outcomes.

### 3.2. Adverse Effects with TRPV1-Targeted Treatments

Targeting TRPV1 has not occurred without complication, and the lessons learned from these adverse effects need to be taken into consideration when developing wound therapy. Early challenges to TRPV1 targeting for analgesia were off-target adverse effects like noxious heat sensation and hyperthermia [65], and so channel activator blockade by antagonists was pursued [42]. A potential downside to targeting TRPV1 may be the carcinogenic effects, but the extent of this risk is still under investigation. One preclinical study found that topical application of AMG-9810, a TRPV1 antagonist, increased epidermal growth factor receptor (EGFR) expression and its downstream Akt/mammalian target of rapamycin (mTOR)-signaling pathway in vivo, as well as cell proliferation promotion in Telomerase-immortalized primary human keratinocytes 1 (N/TERT1) cells [66]. This evidence might suggest the chronic blockade of TRPV1 may increase the risk for cancer development. However, a recent examination of this antagonist and two other TRPV1 antagonists (SB-705498 and PAC-14028) in normal human epidermal keratinocytes (NHEK) and HaCat cells found no change in cell proliferation or EGFR/Akt/mTOR signaling pathway protein expression, nor could AMG-9810-treated skins activate the EGFR signaling pathway in TPA-induced papilloma formation [67]. Testing a TRPV1 antagonist for tumorigenicity in a mouse model of skin carcinogenesis, PAC-14028 1.0% cream was well tolerated by female mice after 169 days of topical application and was not carcinogenic in a two-stage carcinogenesis study [68]. The contradictory findings of these studies need to be addressed, but the potential impact on the EGF/Akt/mTOR signaling pathway does need to be taken into account when utilizing TRPV1-targeted therapies in wound healing.

### 3.3. TRPV1 Involvement in Sepsis

In addition to possible carcinogen risks, preclinical studies may provide evidence of TRPV1 functionality playing a role in the development of sepsis. Mice deficient in TRPV1 with the cecal ligation and puncture (CLP) model of sepsis suffered from more severe disease compared to WT mice, including increased peritoneal mononuclear cell apoptosis, decreased neuropeptide-dependent phagocytosis, decreased reactive oxygen species, increased inflammatory mediator levels, and reduced bacterial clearance [69]. The authors concluded that TRPV1 deletion is associated with decreased macrophage-associated defenses and so TRPV1 protects against sepsis damage, possibly influencing the transition from local inflammation to systemic disease [69]. There is also evidence of a shift of TRPV1 function from anti-inflammatory to pro-inflammatory in systemic inflammatory response syndrome (SIRS) and sepsis models as an organism ages, shedding light on another factor to consider when utilizing TRPV1-targeted therapy in older patients. Wanner and colleagues showed that TRPV1 antagonism in young (12 wks) mice resulted in increased LPS-induced mortality, demonstrating an anti-inflammatory role for TRPV1 in SIRS [70], supporting earlier work in the CLP sepsis model [69,71,72]. Accelerated mortality was also seen with CLP in aged animals with TRPV1 deleted compared to wild type littermates [70]. However, when antagonizing TRPV1 or deleting it globally in middle aged mice (43–44 wks), systemic tumor necrosis factor alpha (TNFα) levels were reduced and mortality was delayed and decreased [70], demonstrating a reversal of TRPV1 function with age and the suppressive role of TRPV1 on TNFα. This evidence reveals a potential adverse effect broad TRPV1 antagonism could have in older individuals, potentially making them susceptible to the development of SIRs or sepsis. This is especially important to consider when treating patients with chronic wounds, who tend to be older individuals and may have concomitant wound infection. The evidence of off-target risks and age-associated function demonstrates that, when developing therapy to improve wound healing, the cell population(s), TRPV1 type and expression levels, and age of patient should be well defined, and potential risks considered. Topical treatment of skin wounds may circumvent these concerns.

## 4. TRPV1 in Skin

### 4.1. Targeting TRPV1 in Skin

One strategy for treating skin pain is targeting expression of TRPV1 on keratinocytes based on the findings of studies in other cell types. A recent report shows in human embryonic kidney (HEK) 293 cells and DRGs, TRPV1 is constitutively internalized in a clathrin- and dynamin-dependent manner when cyclin-dependent kinase 5 (CDK5) phosphorylates adaptor protein complex 2 (AP2) and then the μ2 subunit of AP2 (AP2μ2) interacts directly with TRPV1 [71]. When TRPV1 internalization was inhibited in DRGs, there was reduced inflammatory thermal hyperalgesia, which the authors suggest as a potential drug target for the clinical treatment of pain [71]. While these treatment mechanisms have not been tested in keratinocytes yet, further studies may substantiate their therapeutic anti-inflammatory potential in keratinocytes in addition to their pain reduction via the DRGs.

TRPV1 antagonists and TRPV1 pathway antagonists have been used or are currently in clinical trials to treat atopic dermatitis (AD) and psoriasis. While preclinical study results were positive, their clinical efficacy is yet to be determined [72]. The antagonist PAC-14028 decreased scratching behavior and attenuated or reversed barrier damage by improving the expression of the epidermal barrier proteins, loricrin or filaggrin [72]. Another approach to limiting TRPV1 action is by using CT327/SNA-120, a tropomyosin receptor kinase A (TrkA) inhibitor, which acts by reducing TrkA sensitization. TrkA is the NGF receptor and a major player in the TRPV1 pain pathway, and targeting it results in the downstream upregulation of TRPV1. Application of CT327 0.05% to psoriatic skin resulted in a reduction of pruritis on the visual analog scale but no change in the objective score over vehicle in a phase IIb clinical trial [73]. Tradipitant, an inhibitor of neurokinin 1/substance P, a neuropeptide released after TRPV1 activation on sensory nerves, ameliorated pruritis though not statistically significant in a phase II clinical trial and the results of another phase II trial for tradipitant in AD-associated treatment-resistant pruritus have yet to be published [72]. VPD-737, antagonist of the substance P receptor, neurokinin 1 receptor (NK-1R), showed reduction in chronic pruritus and was well tolerated in a phase II clinical trial for AD [74]. To better understand how these treatments are acting in the skin, it is imperative to fully characterize TRPV1 expression and function, as well as downstream mechanisms, in the various cells that make up the skin compartment.

### 4.2. TRPV1 Expression and Function in Keratinocytes

The expression of TRPV1 by keratinocytes was characterized many years ago and its function extensively studied. Denda and colleagues first demonstrated the presence of TRPV1 (previously known as VR1) on human epidermal keratinocytes in situ in 2001 [75]. Heat, photoaging, and natural aging have been associated with increased TRPV1 expression by human keratinocytes ex vivo and in vivo [76,77]. This may be due in part to the UV irradiation, which has been shown to enhance Src kinase mediated trafficking of TRPV1 to the cell membrane surface in HaCat cells from intracellular vesicles in 15 min [78]. Taken together, these studies provide strong evidence for the expression of TRPV1 on human keratinocytes.

**Figure 3:**
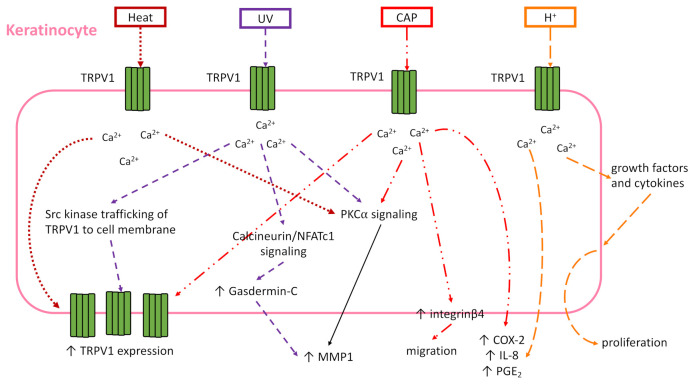
Effects of different activators of TRPV1 on keratinocyte function and signaling. In vitro studies have elucidated TRPV1 is expressed by keratinocytes and activated by several known stimuli. Keratinocyte intra- and extracellular signaling and, in some cases, function was determined post-activation. This figure represents the findings of these various in vitro studies, with different dashed lines showing the downstream signaling/function each activator induced. The signaling shown may or may not occur simultaneously, and this figure demonstrates the overlap in signaling outcomes of the various stimuli. Heat—>43 °C, UV—ultraviolet irradiation, CAP—capsaicin, H^+^—acidic environment.

While keratinocytes may express TRPV1, the role that TRPV1 manipulation plays in the functionality of keratinocytes is complicated, summarized here and in Figure 3, and requires more study. In zebrafish keratinocytes, cell motility and intracellular calcium activity are TRPV1-dependent [79]. This finding was supported in murine keratinocytes (Pam212 cells) which revealed the de novo expression of integrin β4 and TRPV1 in migrating cells, capsaicin promotion of migration, and TRPV1 antagonist inhibition of wound closure in a scratch wound assay [80]. In contrast, it is possible that TRPV1 channel opening by capsaicin or heat may delay barrier recovery after tape stripping in hairless mouse skin and human skin [81]. The detrimental effect of TRPV1 activation finding was also supported recently in nitrogen mustard (NM) skin injury model. Though calmodulin signaling is known to desensitize TRPV1 after activation [46], in vitro NM increased TRPV1 expression, intracellular Ca^2+^, and Ca^2+^/calmodulin-dependent kinase kinase β/AMP-activated protein kinase/unc-51-like kinase 1 (CaMKKβ-AMPK-ULK1) signaling in keratinocytes [82]. This signaling led to autophagy and cell death, and this phenotype was confirmed in vivo in NM-treated skin [82]. TRPV1 activation with trichloroacetic acid (TCA) induced keratinocyte production of growth factors and cytokines that enhance proliferation in vivo [83]. Although it seems TRPV1 activation promotes keratinocyte proliferation in acidic conditions, evidence suggests TRP channels other than TRPV1 are important for keratinocyte differentiation [84,85]. Therefore, it may be better to target a different TRP channel if the desired effect is keratinocyte differentiation instead of proliferation.

In addition to its part in keratinocyte function, TRPV1 significantly impacts keratinocyte-dependent inflammation. TRPV1-dependent inflammation was demonstrated in vitro with NHEK and HaCat cells, where TRPV1 activation by capsaicin or acidification induced increased intracellular calcium and release of cyclooxygenase-2 (COX-2), interleukin (IL)-8 and prostaglandin E_2_ [86,87]. TRPV1 mediated the expression of matrix metalloproteinase 1 (MMP-1) mRNA and protein via calcium influx in response to heat, capsaicin, or UV irradiation in NHEK and HEK 293 cells, and follow-on studies showed the calcium influx resulted in PKCα signaling in HaCat cells for this outcome [78,88,89]. Additionally in HaCat cells, UV radiation activated TRPV1 to open and allow calcium influx, which turned on the calcineurin/nuclear factor of activated T-cells, cytoplasmic 1 (NFATc1) pathway [90]. This in turn induced gasdermin-C expression in the HaCat cells, which also fed into the increase of MMP-1 expression [90]. Taken together, these studies show the various downstream signaling pathways that are activated after TRPV1 opens and allows calcium influx into keratinocytes. Depending on the type of wound being treated, it is important to understand if TRPV1 is activated by injury, and if so, how it affects the functionality of the keratinocytes in wound healing in order to properly target TRPV1 with either agonist or antagonist (Table 1).

### 4.3. TRPV1 Expression and Function in the Pilosebaceous Unit

**Figure 4:**
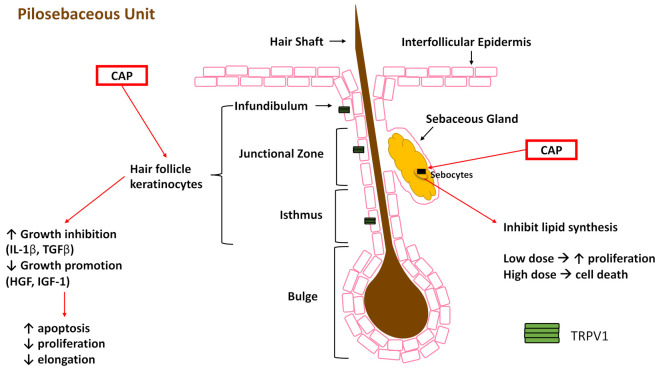
Impact of capsaicin (CAP) on substructures of the pilosebaceous unit. Keratinocytes and their stem cell precursors express TRPV1 (green tetramer, not to scale) and, when stimulated with CAP, show increased expression of growth inhibition factors and decreased growth promotion factors, accompanied by increased apoptosis. CAP activation inhibits lipid synthesis in the sebaceous gland (SG). Additionally, low dose CAP stimulation in the SG has increased proliferation, but high dose CAP stimulation leads to cell death.

The pilosebaceous unit consists of a hair follicle (bulge, isthmus, junction zone, and infundibulum) and a sebaceous gland, each with distinct stem cell populations that remain compartmentalized (Figure 4) and do not contribute to interfollicular epidermis (IFE) during homeostasis [97]. While stem cells of the IFE are responsible for the majority of IFE repair [98], Page and colleagues demonstrated that stem cells from both the hair follicle and sebaceous gland break compartmentalization and contribute to repair of the IFE after wounding [97], supporting earlier work in this field [99,100,101,102]. Moreover, in a murine study, wounds healed significantly faster if the hair follicles were in the anagen (growth) phase of the hair cycle [103], though this enhancement appears to be from the isthmus, infundibulum, and sebaceous gland stem cells, but not the bulge stem cells [104]. Though no studies have shown TRPV1 activation in bulge stem cells contributing to functionality, TRPV1^+^ sensory neurons innervating the hair follicle promoted epithelial proliferation and bulge stem cell progeny migration after wounding [105]. The authors suggested that sensory innervation modulated hair follicle (HF) stem cell physiology through the neuropeptides substance P and CGRP, as receptors for those proteins were seen on CD34^+^ cells of the bulge stem cell niche [105]. The contribution of stem cells from the pilosebaceous unit to re-epithelialization makes it important to understand both the presence of TRPV1 and potential outcome of TRPV1 activation in these cell populations (Figure 4) to better understand how they may contribute during healing.

TRPV1 is present and functional in human and mouse HF and its activation may inhibit hair growth. Bodó and colleagues demonstrated TRPV1 expression in the outer root sheath (ORS), matrix, and, to a lesser degree, the inner root sheath keratinocytes of the human HF in vivo, but TRPV1 was absent from HF mesenchyme and dermal papilla fibroblasts [106]. In an organ-cultured human scalp HF and cultured human ORS keratinocytes, TRPV1-specific stimulation by capsaicin inhibited hair shaft elongation, suppressed proliferation, enhanced apoptosis, led to premature catagen, upregulated hair growth inhibitors (IL-1β and transforming growth factor-β_2_) and downregulated hair growth promoters (hepatocyte growth factor, insulin-like growth factor-I (IGF-I), and stem cell factor) [106]. To advance these findings, analysis of TRPV1 immunoreactivity during depilation-induced HF cycling in mice showed the strongest signal in the regressing catagen and telogen follicles, whereas highly proliferating follicle cells had reduced signal [107]. Additionally, Trpv1^−/−^ mouse skin showed no obvious skin abnormalities compared to wild-type control, however, catagen retardation was observed in the transition from HF morphogenesis to cycling skin appendage [107]. Both cultured human and mouse HF demonstrated similar responses to capsaicin treatment, showing the role of TRPV1 in the hair cycle, and suggesting the detrimental effect activation of TRPV1 on keratinocytes could have on the contribution of hair follicle stem cells to wound healing in the epidermis. By contrast, the stimulation of TRPV1 on subcutaneous sensory nerves with capsaicin induced the release of CGRP, which, in conjunction with isoflavone, increased the IGF-I levels in the skin and hair regrowth of mice [108]. These results were translated into human patients when 48 volunteers with alopecia were orally administered capsaicin and isoflavone for five months and 64.5% of them had higher IGF-I serum levels and significantly promoted hair growth [108]. Taken together, these studies show the potential contradictory effects TRPV1 activation may have in the wound healing environment. If TRPV1 activation on hair follicle keratinocytes leads to apoptosis and hair growth delay while sensory nerve TRPV1 activation induces CGRP release and healing promotion, then these two actions may cancel out each other or result in less effective skin. Timing of TRPV1 activation is another parameter to consider since wound healing is tightly temporally controlled, and a release of CGRP may be beneficial early in healing but detrimental in the later stages, or vice versa.

The other compartment of the pilosebaceous unit is the sebaceous gland (SG), where TRPV1 expression has been shown in a human SG in situ and in vitro using human sebocyte cell culture model (SZ95 sebocytes) [109]. Activation of TRPV1 in SZ95 sebocytes inhibited lipid synthesis in a dose-, time-, and extracellular calcium-dependent manner (Figure 4) [109]. This may be detrimental to barrier recovery as it has been previously shown that lipid synthesis is initiated after injury and different lipids are synthesized in different phases of healing [110]. Additionally, low-dose capsaicin stimulated sebocyte proliferation, while high-dose treatment led to cell death (Figure 4) [109]. This evidence suggests that targeting TRPV1 may be beneficial for acne and other sebaceous diseases, though its presence in sebaceous glands and response to high dose capsaicin needs to be considered when using TRPV1-targeted treatments in wounds.

### 4.4. TRPV1 in Other Mesenchymal Skin Cells

In an extensive study of TRPV1 immunoreactivity of epithelial and mesenchymal cells in normal human skin in situ, sweat gland epithelium was strongly positive, but melanocytes and connective tissue fibroblasts were both found to be TRPV1 negative [111]. In contrast, cultured primary human melanocytes were positive for TRPV1 expression by immunocytochemistry, and were able to increase intracellular calcium when activated by capsaicin, though this did not impact melanogenesis [112]. One possible explanation for these incongruous results may be that the low levels in vivo required more amplification to detect using IHC, as newer technologies have shed light on TRPV1 expression in melanocytes. A genome-wide transcriptome analysis of human epidermal melanocytes found expression of TRPV1, though it was 75 times lower than TRPV2, which was one of the top 25 highly expressed ion channel genes [113]. Kim and colleagues were able to show mRNA and protein expression of TRPV1 in cultured human skin fibroblasts, as well as channel functionality when treated with capsaicin [114]. In murine embryonic fibroblasts, Bode and colleagues found that, not only was TRPV1 expressed, but TRPV1 induced the ubiquitylation of EGFR, leading to EGFR degradation via the lysosomal pathway and reduced malignant cell transformation [115]. While evidence suggests that there may be expression of TRPV1 on melanocytes and fibroblasts, the extent to which it impacts function of these cell populations requires further study, and future studies should shed light on the potential impact of TRPV1-targeted therapy on mesenchymal cells.

### 4.5. TRPV1 in Immune Cells Involved in Wound Healing

The tissue resident immune cells and skin immune responses have been extensively reviewed over the years, including several recent comprehensive reviews [116,117,118] that integrate all immune cells rather than focus on specific cell types or diseases. The multiple immune processes contributing to wound repair are reviewed in this journal issue (and elsewhere [119]), and here we focus on the contribution of the TRPV1 channel to this process. While the utility of many immune cells has been elucidated over the years, the study of TRPV1 function on different immune cells is limited and for the most part has not been conducted in the wound healing environment.

TRPV1 is expressed by mouse mast cell (MC) lines and bone marrow derived MCs, and these cells have demonstrated similar calcium influx and desensitization after TRPV1 activation by capsaicin and RTX [120]. However, there is conflicting evidence about TRPV1 expression on human skin MCs [121,122]. Neutrophils isolated from healthy human anticoagulated venous blood expressed TRPV1, but application of capsaicin failed to induce a current [123]. TRPV1 has been shown to be expressed by murine dendritic cells (DCs) with activation by capsaicin leading to maturation of immature DCs in vitro and migration of dermal DCs to lymph nodes in vivo [124]. In contrast, while human monocyte-derived DCs expressed TRPV1, capsaicin activation inhibited cytokine-induced DC differentiation, DC activation, pro-inflammatory cytokine secretion, and bacterial phagocytosis, suggesting an anti-inflammatory role for TRPV1 on human DCs [125]. Anti-inflammatory actions were also shown in vitro in RAW 264.7 macrophages when capsaicin activation of TRPV1 inhibited LPS-induced NO, iNOS, COX-2 and PGE_2_ production via the NFκB pathway and inhibited IFNγ-induced NO and iNOS via the signal transducer and activator of transcription (STAT)-1 pathway [126]. CD4^+^ T cells, both mouse and human, expressed functional TRPV1, which co-located with the TCR and, in a T cell-mediated colitis model, contributed to TCR-induced IFNγ, IL-17A, IL-2, IL-10, IL-4, and TNFα production [127]. Similar results were found in the allergic rhinitis (AR) mouse model where Th2 and Th17 cytokines were reduced in the TRPV1 knockout AR mice compared to wild type AR mice [128]. Several immune cells express TRPV1, however, differences in functionality have been demonstrated between mouse and human immune cells, and both pro-inflammatory and anti-inflammatory downstream effects are possible depending on the cell and model used.

## 5. Using TRPV1-Targeted Therapy to Improve Wound Healing

While there is strong evidence of the presence of non-neuronal TRPV1 and its many roles in skin function, the evidence for targeting TRPV1 for skin wound healing has proven complicated. The sometimes discrepant results of TRPV1 activation, inhibition, or ablation in various skin diseases (Table 1) and wound types (Table 2) are summarized, and may be due to activator/inhibitor dosage levels, TRPV1 splice variant, animal model, tissue investigated, etc. Despite keratinocytes, fibroblasts, lymphocytes, and vascular endothelial cells in the rat dorsal paw and plantar skin having TRPV1 immunopositivity, chemical treatment with RTX did not influence TRPV1 mRNA and protein expression levels in non-neural cells of either skin types as it does in sensory neurons [39]. This puzzling result may be explained by an earlier study that found HaCat and NHEK cells did not respond to capsaicin or RTX with calcium cytotoxicity like neurons [129], showing that TRPV1, though present on these cells, may not impact their function as much as neurons. The authors suggest that TRPV1B expression, a dominant negative splice variant, and the low level of TRPV1 expression contributed to human keratinocyte resistance to capsaicin and other vanilloids [129]. This may suggest the need for a more personal medicine approach where TRPV1-targeted therapies to improve wound healing are tailored to the channel splice variant expressed and dosage selected based on expression level, as seen in sebocytes, to reach the desired outcome.

### 5.1. TRPV1^+^ Peripheral Sensory Nerves in Skin

There is evidence of TRPV1-targeted therapy impacting the functionality of TRPV1^+^ peripheral sensory nerves in the skin, which may be utilized to improve wound healing. TRPV1^+^ peripheral sensory nerves are known to contribute to vasodilation in the skin through the release of CGRP [141]. Systemic stimulation with RTX was shown to induce sensory neurons in the skin to release pituitary adenylate cyclase activating polypeptide (PACAP-38) [142], and later this same group showed that PACAP regulated TRPV1-mediated acute neurogenic edema in plantar skin after capsaicin injection, though this edema was not accompanied by immune cell infiltration as seen with CFA-induced edema [143]. Poitras and colleagues found that high dose application of TRPV1 agonist capsaicin to neurons in vitro led to toxic neurodegeneration, but low dose capsaicin led to outgrowth plasticity [144]. In vivo, capsaicin treatment of sciatic nerve crush injury resulted in better thermal sensation recovery and improved epidermal axon reinnervation of the skin approaching contralateral skin levels [144]. Local application of capsaicin to mouse ear skin yielded early axon loss followed by later hyperinnervation [144]. This evidence demonstrates the role that TRPV1 activation plays in regeneration of adult sensory neurons and their axons, which may impact reinnervation of wounded skin by thermosensitive axons.

**Figure 5:**
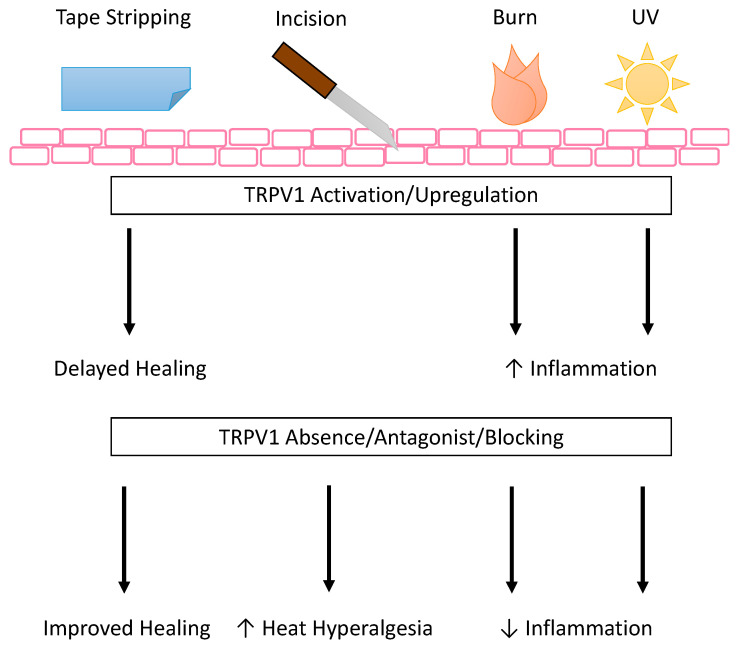
TRPV1 role in different types of wounds. In vivo and ex vivo studies have looked at the outcomes of various types of wounding either in the presence or absence of TRPV1 or with activation or inhibition of TRPV1. These outcomes are summarized in Table 2 and here graphically. Generally, it appears that TRPV1 activation, mainly in keratinocytes, leads to inflammation and delayed healing in tape strip, burn, and UVB wounds. The absence of TRPV1 increases heat hyperalgesia in incision wounds. TRPV1 antagonism or blockade improves healing in tape strip wounds and has an anti-inflammatory effect in burn and UVB wounds.

In addition, TRPV1^+^ nociceptors have been shown to play a role in psoriasis, barrier recovery, AD, and allergic contact dermatitis (ACD). In the imiquimod (IMQ) induced psoriasis model, dermal dendritic cells (dDCs) were imaged in contact with cutaneous sensory neurons expressing TRPV1 and Na_V_1.8 [91]. Further, ablation of these nerves resulted in greatly reduced IMQ-associated inflammation including reduction in immune cell infiltration and cytokine production (IL-23, IL-17A/F, and IL-22) [91]. Using other mouse models, the authors confirmed that TRPV1^+^Na_V_1.8^+^ nociceptors actively induced and controlled IL-23 production by dDCs [91]. In a hairless murine model of tape stripping that previously showed delayed healing in response to capsaicin or heat [81], the TRPV1 antagonist PAC-14028 was shown to accelerate barrier recovery [88,89] (Figure 5). PAC-14028 also alleviated dermatitis-associated damages in the oxazolone-induced AD-like murine model, including reducing IL-4 and IL-13 signaling through STAT-3 and STAT-6 and TRPV1 expression [88,89], aligning with observations of its trial in human AD [145]. In the squaric acid dibutylester (SADBE)-induced ACD model, SADBE not only directly activates the TRPV1 channel but also TRPV1 was expressed on MrgprA3 and MrgprD expressing DRG neurons, and the authors concluded that these TRPV1^+^ neurons were responsible for the persistent itch signal after SADBE exposure [92]. Additionally, TRPV1 deficiency resulted in increased macrophage infiltration and TNFα, IL-1β and IL-6 expression in the SADBE-induced ACD model, providing further evidence of TRPV1^+^ sensory neuron involvement in cutaneous inflammation [92]. In a skin equivalent model, TRPV1 activation by low pH stimulated proliferation when glycolic acid was applied, an effect subsequently blocked by TRPV1 antagonist [146]. These results demonstrate that TRPV1 may be involved in different skin diseases though it may improve or exacerbate depending on the condition, which will determine if it is better to activate or inhibit the TRPV1 channel.

### 5.2. Barrier Tissue Infection

Further evidence for a role of TRPV1^+^ nociceptors in barrier tissues is found in the recent studies of infection in skin and lungs. Chiu et al. showed that TRPV1^+^ DRGs in vitro and Na_V_1.8^+^ nociceptors in vivo, which have 80% overlap with TRPV1^+^ nociceptors [91], are directly activated by pathogen associated molecular patterns (PAMPs), specifically formyl peptides and α-hemolysin [93]. Activated nociceptors released CGRP responsible for modulating the immune response to a subcutaneous injection of *Staphylococcus aureus*. The authors demonstrated this in vitro, where CGRP released by DRG nerves suppressed TNFα production by macrophages [93]. Chiu and colleagues went on to demonstrate the anti-inflammatory effects of TRPV1^+^ nociceptors in the lung in response to *Staphylococcus aureus*. Here, the CGRP released from TRPV1^+^ afferents suppressed recruitment and surveillance of neutrophils, leading to better outcomes for the infected animals due to reduced inflammation-associated damage [94]. The beneficial effect of immunosuppression achieved through TRPV1^+^ sensory nerve ablation was demonstrated in skin also against a different pathogen, *Streptococcus pyogenes* [95].

Not only does TRPV1 play a role in modulating the immune response to bacterial pathogens but work by the Kaplan group and others have extended this to fungal ones as well. In bone infection, *Candida albicans* stimulates Na_V_1.8^+^ nociceptors via Dectin-1 to release CGRP in a TRPV1/TRPA1 dependent manner [147]. CGRP reduces osteoinflammation in response to fungal infection by directly suppressing NFκB p65 via the transcriptional repressor Jdp2 [147]. The Kaplan group found that *C. albicans* directly activated sensory neurons to release CGRP in the skin and the secreted CGRP drove IL-23 production by CD301b^+^ dDCs, proliferation of dermal γδ T cells that produced IL-17A, and resistance to cutaneous candidiasis [96]. Utilizing an elegant optogenetic model system, this group further demonstrated a nerve reflex arc that results in anticipatory innate type 17 immunity at sites adjacent to those stimulated, either by light or infection [148]. Taken together, these studies show that skin nociceptors are directly activated by cutaneous invading microbes and/or microbial products and then communicate with tissue resident immune cells, however, the outcome of this communication, either stimulatory or regulatory, is tissue and context dependent.

### 5.3. Incision Wounds

One of the earliest pieces of evidence that TRPV1^+^ nociceptors were involved in the wound environment was the TRPV1-dependent increase in heat hyperalgesia in a mouse hind paw incision model [149] (Figure 5). This was not mediated by a change in TRPV1 expression, as equivalent expression was shown on the ipsilateral peripheral TRPV1^+^ sensory nerves and the DRG and sciatic nerves as compared to the contralateral skin and nerves [149]. This response was later determined to be dependent on a very specific set of afferents [130], which were also positive for isolectin B4 (IB4) [150], possibly sex dependent [151], and used the IGF-I/Akt signaling pathway to execute their effect [152]. In a later study of the role of TRPV1 in tissue swelling after surgical incision, the loss of TRPV1 did not show a significant difference in hind paw swelling after incision, suggesting TRPV1 does not play a part in incision-associated edema [131]. Taken together, these studies show that the pain associated with incision wounds is dependent on TRPV1^+^ afferents but the edema uses another pathway suggesting a combination therapy may be more beneficial when treating this type of wound.

### 5.4. Burn Wounds

TRPV1 expression and activation seem to be affected by burn injury, known to be a highly inflammatory process, and in turn affect recovery by contributing to the post injury inflammation in both central and peripheral mechanisms. Recent evidence suggests that burn injury increased CGRP-associated TRPV1 channel expression on the DRG [132]. CGRP can induce either pro- or anti-inflammatory responses, depending on context, and in the burn injury model, induced inflammation in DRG nerves [132]. The authors additionally showed that this TRPV1 expression was attenuated by the overexpression of Fibulin-5, an extracellular matrix protein known to induce granulation tissue formation and promote wound healing, by downregulating α subunit of eukaryotic initiation factor 2 phosphorylation, which reduced CGRP, IL-1β, and TNFα expression [132]. In the periphery, evidence suggests the role of oxidized linoleic acid metabolites in activating TRPV1^+^ sensory nerves [133]. This nociceptor activation increased inflammation in the form of increased leukocyte/macrophage infiltration after burn injury, a process that was dependent on cytochrome P450 metabolism of linoleic acid [133]. In addition to the agonist capsaicin, another example of a natural product modifying TRPV1 expression to treat third degree burns is honokiol, a poly-phenolic compound extracted from various magnolia species, though the exact mechanism by which it alters expression is not fully understood. Treatment with this compound, either intradermally or intraperitoneally was shown to decrease mRNA and protein expression of TRPV1 and the purinergic G protein-coupled receptor P2Y, thereby reducing the inflammation and pain associated with burn wounds in mice [134]. Additionally, keratinocytes from burn scars with pruritis have increased thymic stromal lymphopoietin protein levels when compared to keratinocytes from normal control skin, and this increase is enhanced by TRPV1 or TRPV3 activation [135]. In contrast, severe ulceration resulted after application of TCA to mice lacking the TRPV1 channel in a chemical burn model, suggesting a role for TRPV1 in this type of burn [83]. The authors propose TRPV1 is important for the induction of growth factor and cytokine release in the recovery from chemical burn injury [83]. Taken together, this evidence suggests that activation of TRPV1 in the periphery may be disadvantageous in some burn wounds by exacerbating inflammation (Figure 5), unlike its anti-inflammatory effects centrally shown by Chiu and colleagues. Conversely it may be advantageous in chemical burns by leading to the release of CGRP to induce cytokines and growth factors vital to healing. Thus, the context and the overall inflammatory environment may modulate the effects of TRPV1 activation.

### 5.5. UVB Damage Wounds

In another type of wound caused by UV damage, inhibition of TRPV1 on keratinocytes may help dampen UV-induced inflammation (Figure 5). Inflammatory mechanisms have been targeted in mouse keratinocytes using TRPV1-specific blocker 5′-iodoresiniferatoxin (I-RTX) to reduce UV-induced mRNA and protein expression of MMP-13, MMP-9, MMP-3, and MMP-2, mRNA expression of pro-inflammatory cytokines IL-1β, IL-2, IL-4, and TNFα, and protein expression of COX-2 and p53 [136]. More recently, Chung and colleagues have developed a TRPV1 inhibitory peptide^701–709^ and shown that it inhibited UV-induced MMP (MMP-13 and MMP-9 in mouse and MMP-1 and MMP-2 in human) and pro-inflammatory cytokine expression (IL-6 and TNFα in mouse and IL-6 and IL-8 in human), as well as calcium influx in HaCaT cells, mouse skin, and human skin [137]. Taken together, this evidence suggests that in wounds with hyperinflammation, inhibiting TRPV1 on keratinocytes may decrease the keratinocyte dependent inflammatory signals, decreasing feedback and reducing overall inflammation.

One recent study explored the role of neuropeptide, namely CGRP, in sterile wounds, although the work did not specifically address the TRPV1 channel. One high dose of UVB radiation induces inflammation, as evidenced by edema and sensitivity to mechanical stimuli (testing the skin with von Frey filaments). This inflammation is associated with increased infiltration of neutrophils and monocyte-derived macrophages and DCs on days two and three post irradiation followed by increased αβ T cells and dDCs on days 5 and 10 and Langerhans cells on day 10 [153]. The infiltration of T cells and dDCs is reduced when CGRP is knocked out of the mouse, suggesting that CGRP may play a role in the immune cell expansion in response to UVB induced inflammation [153]. Interestingly, using a series of loss of function models, the authors did not find TRPV1 or TRPA1 contributed to this reduced infiltration indicating a mechanism that does not involve these channels [153]. This is a narrow example of UVB wounding and does not exclude the involvement of TRPV1 in low dose UVB wounds, as detailed in the studies noted above, or other TRP channels. However, it does shed light on the role of CGRP in immune cell infiltration after wounding, which may be induced in other models of wound healing via TRPV1 activation, as shown in infection.

### 5.6. Corneal Wounds

Cornea alkali burn causes severe inflammation and tissue fibrosis. However, Okada and colleagues found reduced opacification and stroma thickness, both measures of scarring, in corneas lacking TRPV1, as well as decreased inflammatory mediator gene expression [138]. This phenotype was reproduced with the application of TRPV1 antagonists in the corneas of WT mice, confirming the involvement of TRPV1 in the alkali burn-associated inflammation and fibrosis [138]. In contrast, Sumioka et al. [139] showed the importance of TRPV1 in the healing of epithelium debrided corneal defects in both rat and mouse models. Wounds in rat corneas cultured ex vivo healed defects faster post activation of TRPV1, while blocking its activation delayed healing [139]. In vivo, the absence of TRPV1 in mice was associated with impaired corneal healing, decreased proliferation of epithelium, and reduced IL-6 and substance P gene expression [139]. These findings were further supported in a cornea incision mouse model, which showed reduced collagen type 1a on day 3, delayed TGFβ expression by 5 days compared to the WT control mice, and delayed healing in the Trpv1^−/−^ corneas compared to the WT [154]. Despite these differences, there were no alterations found in infiltrating immune cells [154]. Therefore, in addition to the robust evidence that TRPV1 activation is important for immune signaling in infected wounds, TRPV1 impacts wound healing in the cornea without an immune component. There are many parallels between skin and cornea healing such as inflammatory cell influx, myofibroblast differentiation, extracellular and scar formation [140]. However, unlike the skin where it is essential, the cornea does not induce capillary sprouting after injury [140]. Additionally, the cornea is an immune privileged site resulting in a different immune response if antigen is present during repair [140]. These two important differences need to be taken into account when translating experimental evidence between the cornea and skin for wound healing, as well as the type of wound that is being treated.

## 6. Conclusions

TRPV1 is a nonspecific ion channel highly expressed by cutaneous sensory nerves and other skin cells, including circulating and skin resident immune cells. Understanding the role of TRPV1 in wound healing may inform future TRPV1-targeted therapies to improve healing in impaired and chronic wounds. Many factors contribute to the polymodal nature of TRPV1 channel activation including tetrameric composition, splice variant, accessory protein sensitization or desensitization, activator concentration/coupling, etc. TRPV1 targeting is further complicated by the downstream pathways induced after its activation and the resulting functionality of the cell expressing it, which may lead to adverse or off-target effects, as well as potential shifts in functional outcomes with age. TRPV1-targeted therapies in use and in clinical trials, mainly composed of capsaicin-based treatments, are for pain relief, however, this work has been expanded into the fields of urology, oncology, and dermatology, including skin wound biology.

In skin, TRPV1 is expressed by keratinocytes, sebocytes, nociceptors, and several immune cells, and possibly melanocytes and fibroblasts. Therefore, TRPV1-targeted therapy may impact these cell populations. The preponderance of evidence indicates that TRPV1 expression and activation on keratinocytes seems to promote inflammation. Perhaps in accord with this, skin diseases and wound types that are highly inflammatory benefit from the inhibition or absence of TRPV1 (Table 1 and Table 2). On the other hand, skin acid burn, cornea debridement, and cutaneous fungal infection showed improved outcomes when TRPV1 is activated on epithelium or nociceptors. These TRPV1^+^ nociceptors have been shown to promote Type 17 inflammation in some models and anti-inflammatory mechanisms in other models. These effects are contradictory but likely responsible for balancing inflammation in response to injury or infection to reduce inflammation-associated tissue damage. Therefore, blocking TRPV1 may improve wound healing in inflammatory wounds, though the wound environment must be taken into account when deciding to employ this treatment.

Use of TRPV1-targeted therapy may impact the pilosebaceous unit as well, which may improve or delay healing depending on the dose and timing of treatment. TRPV1 activation on keratinocytes and the sebaceous gland may be detrimental to healing as evidence has shown hair growth inhibition and decreased lipid synthesis. By contrast, TRPV1^+^ nociceptors that innervate the hair follicle play a role in bulge stem cell proliferation and migration to improve healing. Therefore, it is critical to tailor the TRPV1-targeted therapy to extract the most benefit from the pilosebaceous unit for wound healing.

It is clear TRPV1 is expressed in many cell populations in the skin that are involved in wound healing, and activation or inhibition of TRPV1 impacts the function of these cells. However, more work is needed to confirm the benefits of TRPV1-targeted therapies for healing various types of wounds, as well as resolve the dose and timing of these treatments.

## Figures and Tables

**Table 1:** TRPV1 involvement in skin disease.

Condition	Model System	TRPV1 Action	Outcomes	Conclusion	Reference
Psoriasis	IMQ-induced psoriasis in mice	Ablation of TRPV1^+^ nociceptors	TRPV1^+^Na_V_1.8^+^ nociceptors actively induced and controlled IL-23 production by dDCs	Removing TRPV1^+^ nociceptors reduced IMQ-associated inflammation including immune cell infiltration and cytokine production (IL-23, IL-17A/F, and IL-22)	[91]
Nitrogen mustard skin injury	Keratinocytes in vitro and in vivo (SKH-1 hairless mice)	Nitrogen mustard (NM) application to dorsal skin pre-treated with chloroquine (CQ), capsazepine (CPZ), or nothing (NM)	NM activated the TRPV1 signaling through CaMKKβ-AMPK-ULK1 which led to increased inflammatory response and autophagy in skin. Both CQ and CPZ attenuated this response.	NM-induced autophagy is mediated by activating the TRPV1 signaling pathway and causes cutaneous injury	[82]
Atopic dermatitis	Oxazolone (Ox)-induced AD-like murine model	TRPV1 inhibition with PAC-14028	Reduced IL-4 and IL-13 signaling through signal transducer and activator of transcription (STAT)-3 and STAT-6 and reduced TRPV1 expression	Blocking TRPV1 alleviated dermatitis-associated damages	[88,89]
Allergic contact dermatitis	SADBE-induced ACD in WT vs. TRPV1 KO mice	Lack of TRPV1 in KO mice	SADBE directly activated TRPV1 and TRPV1 KO mice had increased macrophage infiltration and TNFα, IL-1β and IL-6 expression	TRPV1^+^ sensory neurons are responsible for persistent itch in SADBE-induced ACD and TRPV1 presence was anti-inflammatory	[92]
Barrier tissue infection	Intradermal injection of *Staphyloccocus aureus*	PAMP activation of TRPV1^+^ nociceptors	CGRP released from TRPV1^+^ nociceptors reduced TNFα production from macrophages	Cutaneous neuronal signaling had an anti-inflammatory effect in Gram-positive bacterial infection	[93]
	*Staphyloccocus aureus* lung infection	Ablation of TRPV1^+^ nociceptors	CGRP suppressed recruitment and surveillance of neutrophils and reduced inflammation-associated damage	Immunosuppression improved infected animal survival	[94]
	Intradermal injection of *Streptoccocus pyogenes*	PAMP activation of TRPV1^+^ nociceptors	PAMPs activated TRPV1^+^ nociceptors to release CGRP that inhibited neutrophil recruitment and function	BonT/A or CGRP antagonism blocked neuron-mediated suppression of host defense preventing infection	[95]
	Topical *Candida albicans* skin infection	PAMP activation of TRPV1^+^ nociceptors	CGRP drove IL-23 production by CD301b^+^ dDCs and proliferation of dermal γδ T cells that produced IL-17A	Cutaneous neuronal signaling had a pro-inflammatory effect in fungal infection	[96]

**Table 2:** TRPV1 involvement in skin wound healing.

Condition	Model System	TRPV1 Action	Outcomes	Conclusion	Reference
Tape stripping	Hairless murine model	TRPV1 activation with capsaicin or heat	Delayed barrier recovery	TRPV1 involved in barrier recovery	[81]
	Hairless murine model	TRPV1 inhibition with PAC-14028	Accelerated barrier recovery	Blocking TRPV1 improved healing	[88,89]
Incision wound	Mouse hind paw incision in WT vs TRPV1 KO mice	Lack of TRPV1 in KO mice	Increased heat hyperalgesia after incision in KO animals	Incision-associated heat hyperalgesia is TRPV1 dependent	[130]
	Mouse hind paw incision in WT vs TRPV1 KO mice	Lack of TRPV1 in KO mice	No difference in edema between WT and KO	Incision-associated edema is TRPV1 independent	[131]
Burn wound	Dorsal skin burn in mouse	Increased TRPV1 expression after burn	CGRP induced inflammation in DRG which was attenuated by Fibulin-5 overexpression through decreased eIF2α phosphorylation	Burn induced TRPV1 expression in CNS which increased inflammation and reduced recovery	[132]
	Partial thickness skin burn in rat	OLAM activation of TRPV1 or pharmacological blockade of TRPV1	Increased OLAM levels in intact skin after burn Increased leukocyte/macrophage infiltration and colocalized with TRPV1-positive fibers after burn Blockade of TRPV1 or OLAM reduced thermal allodynia	Postburn allodynia is dependent on OLAM activation of TRPV1	[133]
	3rd degree burn in mice	Intradermal or intraperitoneal application of honokiol	Decrease mRNA and protein expression of TRPV1 and the purinergic G protein-coupled receptor P2Y	Honokiol treatment reduced inflammation and pain	[134]
	Human keratinocytes from burn with pruritis	TRPV1 activation with capsaicin	Enhanced TSLP production by keratinocytes	TRPV1 activation increased keratinocyte contribution to inflammation	[135]
	TCA-induced chemical burn in WT vs TRPV1 KO mice	Lack of TRPV1 in KO mice	Severe ulceration in KO mice compared to WT	TRPV1-dependent production of growth factors and cytokines was important for chemical burn recovery	[83]
UVB wound	UV irradiated mouse keratinocytes	TRPV1-specific blocker 5′-iodoresiniferatoxin (I-RTX)	Reduced MMP-13, MMP-9, MMP-3, MMP-2, IL-1β, IL-2, IL-4, TNFα, COX-2, and p53 expression	TRPV1 block in keratinocytes had an anti-inflammatory effect after UV irradiation	[136]
	HaCaT cells, mouse skin, and human skin	TRPV1 inhibitory peptide^701–709^ (TIP)	Reduced UV-induced MMP expression (MMP-13 and MMP-9 in mouse and MMP-1 and MMP-2 in human), pro-inflammatory cytokine expression (IL-6 and TNFα in mouse and IL-6 and IL-8 in human), and calcium influx	TRPV1 inhibition in keratinocytes had an anti-inflammatory effect after UV irradiation	[137]
Corneal wound	Mouse cornea alkali burn wound	Lack of TRPV1 in KO mice	Increased scarring and inflammatory mediator gene expression in the presence of TRPV1	TRPV1 contributes to inflammation and fibrosis in cornea burn healing	[138]
	Rat cornea debridement wound	Activating TRPV1	Accelerated wound healing	TRPV1 promotes healing in the rat cornea	[139]
	Mouse cornea debridement wound	Inhibiting TRPV1	Delayed wound healing	TRPV1 promotes healing in the mouse cornea	[139]
	Mouse cornea debridement wound in WT vs. TRPV1 KO mice	Lack of TRPV1 in KO mice	Impaired healing, decreased epithelial proliferation and migration, and reduced IL-6 and substance P gene expression in KO mouse corneas	TRPV1 signal promotes healing in the mouse cornea including IL-6 and substance P upregulation	[139]
	Cornea incision wound in WT vs. TRPV1 KO mice	Lack of TRPV1 in KO mice	Reduced collagen type 1a on day 3, delayed TGFβ expression, and delayed healing in the KO corneas	Lack of TRPV1 in mouse cornea is associated with reduced pro-healing biomarkers and delayed healing	[140]

## Data Availability

Not applicable.
